# Association of radiotherapy with preferential depletion of luminal epithelial cells in a *BRCA1* mutation carrier

**DOI:** 10.1186/2162-3619-1-31

**Published:** 2012-10-08

**Authors:** Huai-Chin Chiang, Sreejith J Nair, I-Tien Yeh, Alfredo A Santillan, Yanfen Hu, Richard Elledge, Rong Li

**Affiliations:** 1 Department of Molecular Medicine, University of Texas Health Science Center at San Antonio, San Antonio, TX, 78245, USA; 2 Department of Medicine, Cancer Therapy and Research Center, University of Texas Health Science Center at San Antonio, San Antonio, TX, 78245, USA

**Keywords:** Radiation, Luminal progenitor cells, Cell of origin, BRCA1

## Abstract

Radiation therapy (RT) after breast conservation therapy has recently been linked with significant reduction in risk of ipsilateral breast cancer among *BRCA1* mutation carriers. However, the exact mechanism by which RT reduces incidence of *BRCA1*-associated cancer remains unclear. Here we studied fresh breast tissue from a *BRCA1* mutation carrier who was initially treated with a lumpectomy and RT for a unilateral cancer and two years later chose a prophylactic bilateral mastectomy while remaining cancer-free. Flow cytometry analysis demonstrated a strikingly lower luminal cell population in the irradiated breast as compared to the non-irradiated breast, which was confirmed by immunohistochemistry. Furthermore, the irradiated breast tissue exhibited very low progenitor cell activity *in vitro*. Given the emerging evidence that *BRCA1* tumors originate from luminal progenitor cells, our observations suggest that preferential and long-lasting elimination of luminal ductal epithelium may partly underlie the mechanism of RT-associated reduction in recurrence of *BRCA1*-associated cancer.

## Background

Women who carry cancer-predisposing germ-line mutations in *BRCA1* have up to 80% chance of developing breast cancer in their lifetime [1,2]. Narod and coworkers recently reported that radiation therapy (RT) was associated with a significant reduction in the risk of ipsilateral breast cancer among *BRCA1* mutation carriers [3]. Although the exact mechanism by which radiation affects breast cancer recurrence is not clear, it has traditionally been thought to be due to killing of residual tumor cells left behind after excision of the primary tumor [4,5].

*BRCA1*-associated tumors usually have the “basal-like” features and therefore had been presumed to originate from basal stem cells. Surprisingly, recent work from several laboratories suggests that *BRCA1* tumors likely originate from luminal progenitor cells [6-8]. Given the well-documented role of BRCA1 in double-strand break repair and radiation sensitivity [9,10], selective elimination of the cell of origin for *BRCA1*-associated tumors could be an additional or alternative mechanism by which RT reduces incidence of ipsilateral breast cancer in *BRCA1* mutation carriers.

In the current study, we analyzed the abundance and activity of luminal epithelial cells from irradiated and non-irradiated breast tissue of a *BRCA1* mutation carrier who underwent bilateral prophylactic mastectomy. Our result supports the notion that precancerous luminal progenitor cells of *BRCA1* mutation carriers are particularly sensitive to radiation. The potential lineage-specific radiosensitivity of cells from *BRCA1* mutation carriers could be exploited to develop novel prophylactic measures for this select group of at-risk women.

## Materials and methods

### Isolation of human breast epithelial cells

Human breast tissue from mastectomy was dissociated enzymatically with collagenase and hyaluronidase per published procedure [11].

### Flow cytometry and FACS-sorting

According to a previously published protocol [6], mammary cell suspension was pre-blocked and then labeled with an allophycocyanin-conjugated rat antibody to CD49f and FITC-conjugated mouse antibody to human EpCAM. Lineage-negative hematopoietic and endothelial cells were labeled with biotin-conjugated mouse antibodies to CD45, CD235a and CD31, followed by pacific blue-conjugated streptavidin. 7-ADD (BD Bioscience) was added to cells before analysis for live/dead cell discrimination. Cell sorting was performed on a FACSAria flow cytometer (Becton Dickinson). Cells were sorted into four fractions as follows: EpCAM^-^ CD49f stromal cells, EpCAM^low^ CD49f^high^ basal epithelial cells, EpCAM^high^ CD49f^+^ luminal progenitor cells, and EpCAM^high^ CD49f^-^ mature luminal epithelial cells.

### Immunohistochemistry

Immunohistochemistry of formalin-fixed paraffin-embedded samples was carried out with a commercially available ADH5 staining kit (Cat. # PM360DSAA,H; Biocare Medical).

### In vitro mammary colony-forming cell assay

Sorted epithelial cells obtained from primary breast tissue were seeded with NIH 3T3 feeder cells as previously described [12]. After culturing for 7–12 days, cells were briefly fixed with methanol and acetone, stained with Wright’s Giemsa; and colonies were visually scored under a dissecting microscope.

## Case presentation

A *BRCA1* mutation carrier initially received a lumpectomy with histologically negative margins and whole-breast RT (5040 cGy in 28 fractions) for a unilateral cancer in the right breast, and was subsequently found to have a cancer-predisposing *BRCA1* mutation (4987C>G). After learning the results of the genetic testing, the patient chose to have a bilateral mastectomy two years after her original treatment, while remaining cancer-free. Upon obtaining an Institutional Review Board (IRB)-approved informed consent, fresh cancer-free tissue from the non-irradiated and irradiated breasts was obtained at the time of surgery and immediately processed in parallel for single-cell isolation [11]. Simultaneous comparison of bilateral breast tissue from the same person allowed us to exclude individual-based variation.

**Figure 1:**
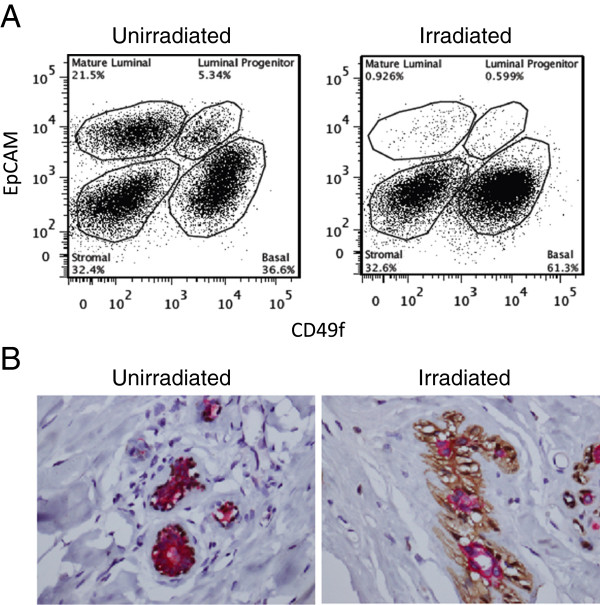
**Irradiated breast tissue has a diminished luminal compartment. A**. Flow cytometry analysis of cancer-free tissue from the non-irradiated and irradiated breasts of a *BRCA1* mutation carrier. (**B**). Immunohistochemistry shows more prominent basal/myoepithelial cells (brown) than luminal cells (red) in irradiated vs. non-irradiated breast glands.

Fluorescence-activated cell sorting (FACS) using established cell surface markers [6,12-14] identified four distinct lineage-negative cell populations, namely, mature luminal epithelial cells (EpCAM^high^CD49f^-^), luminal progenitor cells (EpCAM^high^CD49f^+^), stromal cells (EpCAM^−^CD49^-^), and basal epithelial cells (EpCAM^low^CD49f^high^). The purity of the sorted cells was validated by qRT-PCR analysis of mRNA species that are enriched in stromal (vimentin), luminal (keratin 18), and basal cells (keratin 14) (data not shown). The FACS profile indicated that the previously irradiated breast contained much lower percentages of mature luminal cells (0.926% vs. 21.5%; top right in Figure 1A) and luminal progenitor cells (0.599% vs. 5.34%; top right in Figure 1A) than the non-irradiated breast. This represents a 95% and 89% reduction in these two luminal epithelial cell populations, respectively. Histologic examination shows atrophic lobules in the irradiated breast, in contrast to proliferative fibrocystic changes in the non-irradiated breast. Consistent with the FACS result, immunohistochemistry with a cocktail of antibodies for luminal and basal/myoepithelial markers (ADH5; Figure 1B) indicates a reduced ratio of luminal over basal/myoepithelial cell populations in the irradiated breast (1.422 ± 0.410 (s.d.) for non-irradiated; 0.375 ± 0.155 (s.d.) for irradiated; p = 0.0014).

**Figure 2:**
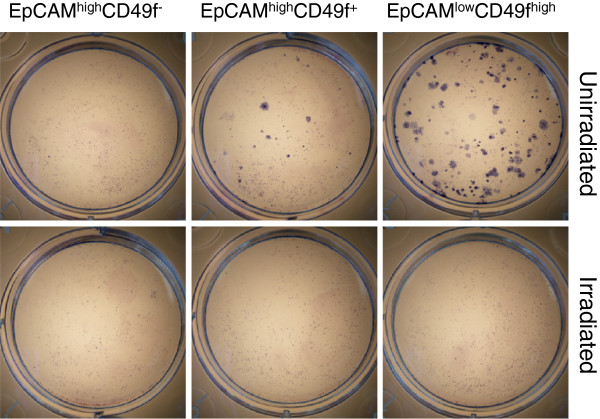
**Reduced progenitor cell activity in the previously irradiated breast tissue.** An equal number of live epithelial cells from the non-irradiated and irradiated breasts were plated together with γ-irradiated NIH 3T3 feeder cells. The images were taken a week after culturing.

Next, we used an established mammary colony-forming cell (Ma-CFC) assay [12], [13] to enumerate the progenitor cells of the sorted epithelial cell populations. An equal number (200/cm^2^; 800 cells/well) of live epithelial cells from the non-irradiated and irradiated breast samples were seeded together with NIH 3T3 feeder cells. Consistent with published results [14], both the EpCAM^low^CD49f^high^ and EpCAM^high^CD49f^+^, but not EpCAM^high^CD49f^-^, fractions from the non-irradiated breast gave rise to cell colonies (Figure 2). In stark contrast, none of the three epithelial fractions from the previously irradiated breast produced any colonies. This result strongly suggests that the previously irradiated breast of the *BRCA1* mutation carrier has substantially diminished progenitor cell activity.

## Discussion and conclusion

RT is associated with reduced recurrence of sporadic [15,16] as well as *BRCA1*-associated breast cancer [3]. In fact, whole-breast low-dose RT has been proposed as an alternative to prophylactic mastectomy [17]. Historically, RT was thought to primarily target residual tumor cells left behind after excision of the primary tumor, despite histologically cancer-free margins [4,5]. While this remains a viable hypothesis, our finding that RT is associated with a dramatically reduced luminal compartment and diminished progenitor cell activity provides an additional possible mechanism by which RT could reduce incidence of cancer recurrence. Our observation is consistent with the notion that radiation reduces the number of non-cancerous luminal progenitor cells, the likely cell of origin for *BRCA1*-associated tumors, thus leading to reduced cancer incidence.

There are several limitations in the current study. First, while our data indicate a relatively larger fraction of basal/myoepithelial cells in the irradiated breast, it is unclear whether this was due to an absolute increase in their number or a relative effect due to the decrease in luminal progenitor cells. However, the similarity in the abundance of the stromal proportion in both breasts argues that there were absolute changes in number of both epithelial compartments. Second, whether the changes observed in our study are unique to breast epithelial cells from *BRCA1* mutation carriers needs to be determined. In addition, it is important to validate these results when more samples of *BRCA1* patients with similarly uncommon confluence of events become available. However, since these results are consistent across disparate technological and methodological platforms, including FACS, IHC, and the cell-based functional assay, it is unlikely that they are due to chance alone. Lastly, we do not know the long-term effects of RT on the breast cancer incidence for the particular individual involved in the study.

The generality of the BRCA1 function in double-strand break repair, as demonstrated in numerous *in vitro* systems, is in stark contrast to its highly tissue-specific nature as a tumor suppressor. Consistent with this, there are no reports of excessive radiosensitivity in skin or connective tissue of *BRCA1* mutation carriers who receive RT. Likewise, we did not observe any significant difference between the stromal cell populations of the irradiated and non-irradiated breast samples. Therefore, radiosensitivity of cells from *BRCA1* mutation carriers may depend on their tissue location, differentiation lineage, and developmental stage. As our study was conducted two years after RT, the observed difference is most likely long-term, not acute, effects of radiation. This is an especially important consideration when exploring a radiation-based preventive approach.

In conclusion, our study reveals an interesting association between RT and reduced luminal epithelial population in the breast tissue of a *BRCA1* mutation carrier. The potential vulnerability of luminal epithelial cells to RT could be the “Achilles’ heel” for the cell of origin of *BRCA1*-associated tumors, exploitation of which may guide the development of novel preventive options for this very select patient population.

## Consent

Written informed consent was obtained from the patient for publication of this Case report and any accompanying images. A copy of the written consent is available for review by the Editor-in-Chief of this journal.

## Abbreviations

FACS: Fluorescence-activated cell sorting; IHC: Immunohistochemistry; IRB: Institutional Review Board; Ma-CFC: Mammary colony-forming cell; qRT-PCR: Quantitative reverse transcription-polymerase chain reaction; RT: Radiation therapy.

## Competing interests

The author(s) declare that they have no competing interests.

## Authors' contributions

H-C. C and S.N. conducted the laboratory experiments including FACS and colony forming assay; I-T. Y. conducted the IHC and analysis; R.E. and A.A.S. recruited the patient and provided clinical guidance and interpretation to the work; Y.H. and R.L. designed the experiments; and R.L. wrote the manuscript. All authors read and approved the final manuscript.

## Authors’ information

Co-First Authors: Huai-Chin Chiang and Sreejith J Nair.
